# Retrospective Analysis of a Real-Life Use of Tixagevimab–Cilgavimab plus SARS-CoV-2 Antivirals for Treatment of COVID-19

**DOI:** 10.3390/ph16101493

**Published:** 2023-10-20

**Authors:** Nicolina Capoluongo, Annamaria Mascolo, Francesca Futura Bernardi, Marina Sarno, Valentina Mattera, Giusy di Flumeri, Bruno Pustorino, Micaela Spaterella, Ugo Trama, Annalisa Capuano, Alessandro Perrella

**Affiliations:** 1 UOC Emerging Infectious Disease with High Contagiousness, AORN Ospedali dei Colli P.O. C Cotugno, 80131 Naples, Italy; nicolina.capoluongo@ospedalideicolli.it (N.C.); marina.sarno@studenti.unina.it (M.S.); giusy.diflumeri@ospedalideicolli.it (G.d.F.); bruno.pustorino@ospedalideicolli.it (B.P.); 2 Campania Regional Centre for Pharmacovigilance and Pharmacoepidemiology, 80138 Napoli, Italy; annamaria.mascolo@unicampania.it (A.M.); micaela.spatarella@ospedalideicolli.it (M.S.); annalisa.capuano@unicampania.it (A.C.); 3 Department of Experimental Medicine—Section of Pharmacology “L. Donatelli”, University of Campania “Luigi Vanvitelli”, 81100 Napoli, Italy; 4 Directorate-General for Health Protection, Campania Region, 80143 Naples, Italy; bernardi.francesca.futura@gmail.com (F.F.B.); ugo.trama@regione.campania.it (U.T.); 5 UOSD Pharmacovigilance, AORN Ospedali dei Colli P.O. C Cotugno, 80131 Naples, Italy; valentina.mattera@ospedalideicolli.it

**Keywords:** COVID-19, tixagevimab–cilgavimab, remdesivir

## Abstract

Tixagevimab–cilgavimab is effective for the treatment of early COVID-19 in outpatients with risk factors for progression to severe illness, as well as for primary prevention and post-exposure prophylaxis. We aimed to retrospectively evaluate the hospital stay (expressed in days), prognosis, and negativity rate for COVID-19 in patients after treatment with tixagevimab–cilgavimab. We enrolled 42 patients who were nasal swab-positive for SARS-CoV-2 (antigenic and molecular)—both vaccinated and not vaccinated for COVID-19—hospitalized at the first division of the Cotugno Hospital in Naples who had received a single intramuscular dose of tixagevimab–cilgavimab (300 mg/300 mg). All patient candidates for tixagevimab–cilgavimab had immunocompromised immune systems either due to chronic degenerative disorders (Group A: 27 patients) or oncohematological diseases (Group B: 15 patients). Patients enrolled in group A came under our observation after 10 days of clinical symptoms and 5 days after testing positivite for COVID-19, unlike the other patients enrolled in the study. The mean stay in hospital for the patients in Group A was 21 ± 5 days vs. 25 ± 5 days in Group B. Twenty patients tested negative after a median hospitalization stay of 16 days (IQR: 18–15.25); of them, five (25%) patients belonged to group B. Therefore, patients with active hematological malignancy had a lower negativization rate when treated 10 days after the onset of clinical symptoms and five days after their first COVID-19 positive nasal swab.

## 1. Introduction

Antiviral therapies, when used alone, have been reported to not always be sufficient to change the course of the COronaVIrus Disease 2019 (COVID-19) [1], especially in frail patients. Therefore, identifying new therapeutic options to prevent or fight this disease in its early phase is essential [2].

Tixagevimab–cilgavimab is a long-acting monoclonal antibody combination of two Fc-modified human monoclonal antibodies obtained from patients who recovered from COVID-19. Tixagevimab and cilgavimab bind non-overlapping sites of the spike (S) glycoprotein of the Severe Acute Respiratory Syndrome COronaVirus-2 (SARS-CoV-2), the causative agent of COVID-19 [3]. The Fc region was modified to extend their half-life (to about 90 days) and reduce binding to the Fc receptor and C1q complement, minimizing the risk of increasing disease inflammation [4,5]. This extended half-life could also offer the advantage of long-term protection against symptomatic COVID-19, compared to the shorter half-lives of other anti-SARS-CoV-2 monoclonal antibodies (approximately 18–32 days) [6,7,8]. Tixagevimab–cilgavimab, by binding to different sites of the S protein, may also help to overcome the immune escape phenomenon and maintain effectiveness against SARS-CoV-2 variants [9]. Based on these beneficial properties, this combination was authorized in Europe for the treatment of early COVID-19 in outpatients (aged ≥ 12 years and weighed at least 40 kg) who do not require oxygen supplement therapy and have risk factors for progression to severe illness, as well as for pre-exposure prophylaxis to SARS-CoV-2 [10]. 

After marketing authorization, a randomized Phase 3 clinical trial was published. This trial compared tixagevimab–cilgavimab with a placebo in hospitalized COVID-19 patients receiving remdesivir and standard care, finding no improvement in the primary outcome (time to sustained recovery), but a good safety profile and a low mortality rate in the tixagevimab–cilgavimab group [11]. Moreover, little evidence has described the use of tixagevimab–cilgavimab in patients affected by hematological malignancies or impaired immune systems [12,13].

Although tixagevimab–cilgavimab seems to be an important therapeutic strategy for protecting people who cannot be vaccinated or who respond poorly to COVID-19 vaccines and for treating early COVID-19, based on the few pieces of real-world evidence available in frail patients, further research is needed to better define the therapeutic role of this medicine. Therefore, we decided to conduct a retrospective observational chart review to describe the use of tixagevimab–cilgavimab in patients affected by COVID-19 who are also affected by other comorbidities, such as hematological malignancies.

## 2. Results

Forty-two patients were enrolled that had received tixagevimab–cilgavimab. All patients were affected by omicron SARS-CoV-2 variants and were hospitalized for causes other than COVID-19, mainly due to comorbidities. No patient complained of side effects related to the administration of tixagevimab–cilgavimab. 

Of the 42 enrolled patients, 21 were female and 21 were male, with a median age of 71 years (Interquartile range, IQR: 78.5–59.0). Fifteen patients had oncohematological diseases (Group B); specifically, 11 patients were affected by active non-Hodgkin lymphoma (NHL) and four patients by chronic lymphocytic leukemia (CLL). A total of 27 patients were affected by chronic disorders (Group A), including cardiovascular disorders (n = 8), degenerative diseases (n = 7), solid tumors (n = 5), infections (n = 4), and autoimmune diseases (n = 3). Ten (37%) patients of group A were affected by more than one chronic disorder. The basal characteristics of these patients are shown in Table 1, while the underlying pathologies are in Table 2. Eleven (40.7%) patients of group A and 12 (80.0%) patients of group B were treated with remdesivir (Table 1). One patient in group B did not receive remdesivir treatment because she was discharged against the advice of the health care workers with oxygen therapy with a Venturi mask (during their short hospitalization, the patient presented a rapid worsening of respiratory function). Patients with NHL and CLL also had immunoglobulin deficiency. Two patients presented with sepsis upon admission to the hospital. One patient had legionella pneumonia. The enrolled patients presented a variegated pulmonary CT picture (Table 3). 

**Figure 1:**
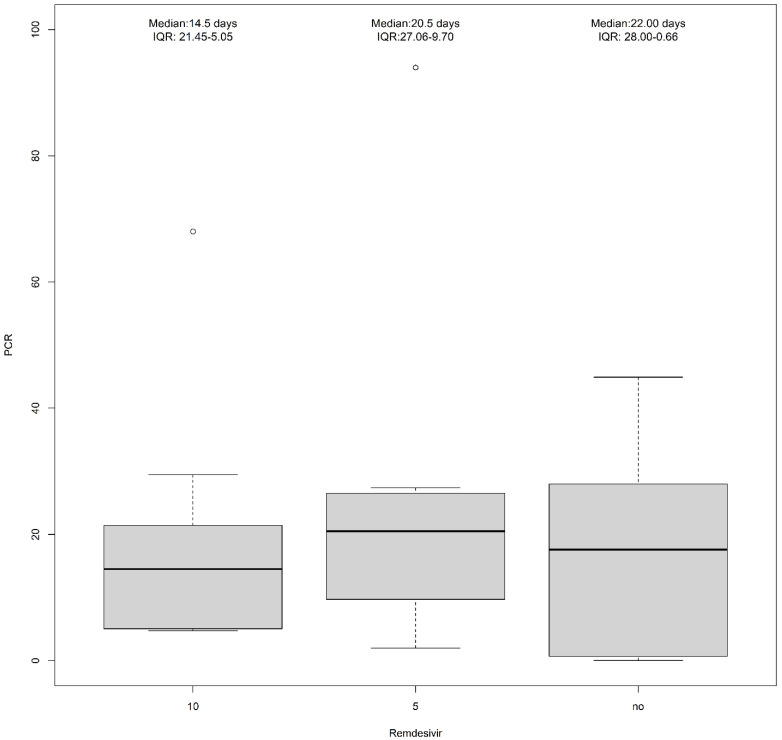
PCR levels according to remdesivir treatment (10 mg, 5 mg, or no treatment).

Of the 42 enrolled patients, 16 patients were unvaccinated (12 patients for group A and 4 patients in group B). IL-6 levels were similar between groups. CRP at admission was higher in Group A compared to Group B (Table 1). Moreover, in stratifying CRP levels for remdesivir treatment, we found higher median levels for 5 mg remdesivir (Median: 20.5; IQR: 27.06–9.70, Figure 1).

The mean stay in hospital of patients in Group A was 21 ± 5 days vs. 25 ± 5 days in Group B. Twenty patients tested negative after a median of hospitalization stay of 16 days (IQR: 18–15.25); of them, five (25%) patients belonged to group B. Eight patients died from COVID-related respiratory failure—four for each group. Two patients in Group B presented with respiratory distress syndrome. Patients enrolled in our study and affected by CLL and NHL came to our observation 10 days after their first clinical symptoms, having tested positive for COVID-19 within 5 days of hospitalization—unlike the other patients enrolled in this study.

## 3. Discussion

Patients with COVID-19 at high risk of being hospitalized or of death—such as older adults, those with multiple comorbidities, or immunocompromised patients—need to be treated early [14,15,16,17]. Specifically, patients with an impaired immune system are at higher risk of prolonged or unresolved SARS-CoV-2 infection, which might also facilitate the development of new variants [18]. In fact, the presence of active malignancy as well as the type of hematological malignancy—together with age, the presence of comorbidities, length of stay in the Intensive Care Unit, and need for mechanical ventilation—are recognized risk factors for adverse outcomes in patients with COVID-19 and hematological malignancies [19]. 

In this study, we observed a similar mean hospital stay between patients treated with tixagevimab–cilgavimab affected by oncohematological tumors and those affected by chronic disorders, despite the higher negativization rate for those affected by chronic disorders (75%). Indeed, patients with hematologic malignancy are characterized by a more compromised immune response to SARS-CoV-2 and high mortality rates (about 34%) [19]. Moreover, hematologic patients can have an impaired response to COVID-19 vaccines by failing in the production of neutralizing and protective anti-S antibodies after a full vaccination cycle [20]. This poor response is common in patients with B cell tumors, such as in CLL [21]. Our hematologic patients mostly had a three-dose schedule of COVID-19 vaccines (n = 10; 66.7%). In the literature, the administration of tixagevimab–cilgavimab did not change the response to COVID-19 vaccines [22], but rather potentiated the pre-existing protection against SARS-CoV-2 infection—even in immunocompromised patients receiving full vaccination [23,24].

Moreover, the low negativization rate observed in patients with hematological tumors may also be due to a delayed start of treatment with tixagevimab–cilgavimab, since patients came to our observation only after 10 days of clinical symptoms in mean and 5 days after testing positive with a COVID-19 nasal swab. This may suggest the importance of early interception in frail patients, with a nasal swab for COVID19 being given when the patient is affected by flu-like symptoms, and then starting early treatment with tixagevimab–cilgavimab to have a higher probability of a good and effective clinical response to the therapy. 

The effectiveness and safety of tixagevimab–cilgavimab as a pre-exposure prophylaxis against COVID-19 has been widely evaluated. A meta-analysis found that tixagevimab–cilgavimab prophylaxis may reduce the rate of SARS-CoV-2 infection (OR: 0.24; 95% CI: 0.15–0.40) and COVID-19 hospitalization (OR: 0.13; 95% CI: 0.07–0.24), and decrease the severity (OR: 0.13; 95% CI: 0.07–0.24) and mortality (OR: 0.17; 95% CI: 0.03–0.99) associated with COVID-19 [25]. Another meta-analysis evaluated the effectiveness of tixagevimab–cilgavimab prophylaxis in immunocompromised participants—including patients with hematological malignancies—confirming the overall clinical effectiveness of tixagevimab/cilgavimab in terms of hospitalization, intensive care admission, and mortality [26]. Both meta-analyses showed the efficacy and safety of tixagevimab–cilgavimab for preventing COVID-19. However, evidence of its efficacy as a post-exposure treatment has been more conflicting. A randomized, double-blind, Phase 3, placebo-controlled trial (ACTIVE-3 study) investigating the efficacy of tixagevimab–cilgavimab compared to placebo in patients treated with remdesivir and other standard therapies found no improvement in the primary outcome of time to sustained recovery with tixagevimab–cilgavimab, but it was safe and showed low mortality [11]. Another Phase 3 study (STORMCHASER study) evaluated treatment with tixagevimab/cilgavimab as a post-exposure prophylaxis against symptomatic COVID-19, finding no difference in the incidence of post-dose positive symptomatic COVID-19 compared to a placebo [27]. On the contrary, a Phase 3, randomized, double-blind, placebo-controlled trial (TACKLE study) demonstrated that tixagevimab/cilgavimab can prevent the development of severe COVID-19 by reducing the risk of severe COVID-19 or death by 50.5% (95% CI 14.6–71.3; *p* = 0.0096) and 66.9% (95% CI 31.1–84.1; 0.0017) in patients with mild or moderate COVID-19 who were symptomatic for less than 5 days, respectively [8]. In particular, this study suggested the efficacy of tixagevimab/cilgavimab in reducing COVID-19 progression and death in high-risk patients [8]. However, it should be highlighted that the most representative risk factors (>10%) were obesity, smoking, hypertension, diabetes, and lung diseases, while the immunocompromised state was underrepresented [8].

We observed a good safety profile for tixagevimab/cilgavimab, in accordance with the results of aforementioned clinical trials in which most events were found to be mild to moderate in severity, with a similar incidence in both the tixagevimab–cilgavimab and placebo groups [8,11,26]. In our study, all patients were affected by omicron variants. In this regard, in-vitro studies have shown the efficacy of tixagevimab/cilgavimab in neutralizing the BA.1, BA.1.1, BA.2, BA.2.12.1, BA.3, BA.4, and BA.5 omicron subvariants, with a potency within the half maximal inhibitory concentration (IC50) range of 4.0–806.0 ng/mL [28,29,30,31]. These results have also been reported in previous studies on the neutralizing activity of monoclonal antibodies for COVID-19 in the treatment of Omicron-infected patients; in particular, combining available mAbs could be an attractive option for targeting newly emerging SARS-CoV-2 variants [32,33].

The main limitation of our study was the small number of patients retrospectively enrolled and treated with tixagevimab–cilgavimab, which also hindered the execution of an adequate statistical analysis. Even so, we described our experience in the use of tixagevimab–cilgavimab in patients with chronic and oncohematological disorders, thus providing new data on the safety and efficacy of this therapy in frail patients affected by Omicron variants, and underlining the possible importance of starting early treatment.

## 4. Materials and Methods

In this observational retrospective chart review study, we enrolled patients who were nasal swab-positive for SARS-CoV-2 (Antigenic and molecular)—vaccinated or not for COVID-19—hospitalized at the first division of the Cotugno Hospital in Naples (UOC of Emerging and Highly Contagious Infectious Diseases), who had received an early therapeutic dose of tixagevimab–cilgavimab from 8 July 2022 to 10 January 2023. This treatment schedule was based on AIFA (Italian Agency for Drug) policy https://www.aifa.gov.it/-/aifa-autorizza-l-utilizzo-terapeutico-del-monoclonale-evusheld-per-il-trattamento-precoce-del-covid-19-in-soggetti-a-rischio-di-progressione, (accessed on 11 September 2023). Basically, patients received an intramuscular single dose of tixagevimab–cilgavimab of 300 mg/300 mg. Patients were divided into two groups: those affected by chronic disorders (Group A) and those affected by oncohematological diseases (Group B). Early treatment and the chronic disorders included were defined according to the Italian ministry of health classification (“Gestione domiciliare dei pazienti con infezione da SARS-CoV-2” aggiornamento del 10 febbraio 2022 in (sanitaria Dgdpsedp, ed), Ministero della Salute, Roma: (2022)—https://www.quotidianosanita.it/allegati/allegato3418644.pdf, accessed on 11 September 2023). Early recognition of positive patients was made possible according to Campania Region BigData SINFONIA [34] and the regional COVID-19 patients early enrolment treatment protocol (https://www.ordinemedicinapoli.it/upload/file/id-0-1657026671-piano%20regionale%20di%20contrasto%20al%20covid-19%20-%20luglio%202022.pdf, accessed on 11 September 2023). 

The two groups were evaluated for their length of stay in hospital (expressed in days) and time elapsed to negativity for COVID-19 at follow-up. Patients’ venous blood samples were analyzed for immunoglobulins A (IgA), M (IgM), and G (IgG); C-reactive protein (CRP); procalcitonin; interleukine-6 (IL6); D-dimer; and fibrinogen, and were retrospectively evaluated during hospital admission every 48 h. High resolution CT scans of the chest (HR chest CT) at hospital admission and hospital discharge were performed too. Antigen testing for SARS-CoV2 on the nasal swab (Methodical: Chemiluminescence Enzyme ImmunoAssay) and viral RNA testing using four gene assays were used to follow-up SARS-CoV-2 evolution and negativizing. 

Positivity tests for anti-spike antibodies were routinely performed, but did not exclude treatment with intramuscular tixagevimab–cilgavimab. 

## 5. Conclusions

In conclusion, patients with active hematological malignancy are those with the worst prognosis for COVID-19, despite therapy with tixagevimab–cilgavimab and remdesivir when treated 10 days after the onset of clinical symptoms and five days after the first COVID-19-positive nasal swab.

These results, according to the new COVID-19 wave currently present in Europe and the USA [35], could be considered in the planning of strategies for early interception in frail patients, aiming to treat them as soon as possible with current antiviral and monoclonal antibodies. This could be made possible with the use of Big Data IT platforms and machine learning algorithms for early identification and direction towards treatment in patients positive for COVID-19, as in SINFONIA [34].

Therefore, it could be useful to encourage hematologists and patients with active hematological malignancies towards the early interception of COVID-19 and to start pharmacological treatment within 5 days after a positive nasal swab. Further studies with an adequate sample size are needed to better elucidate the efficacy and safety of tixagevimab–cilgavimab in patients with COVID-19 and those affected by chronic comorbidities or an impaired immune response.

## Figures and Tables

**Table 1:** The demographic, laboratory, and clinical characteristics of the 42 patients with COVID-19 receiving tixagevimab–cilgavimab. Group A: patients affected by chronic disorders; Group B: patients affected by oncohematological disorders.

	A (N = 27)	B (N = 15)	Overall (N = 42)
**Age**			
Mean (SD)	66.8 (18.2)	69.9 (10.1)	68.0 (15.6)
Median [Min, Max]	71.0 [35.0, 98.0]	73.0 [49.0, 88.0]	71.0 [35.0, 98.0]
Missing	2 (7.4%)	0 (0%)	2 (4.8%)
**Gender**			
F	15 (55.6%)	6 (40.0%)	21 (50.0%)
M	12 (44.4%)	9 (60.0%)	21 (50.0%)
**CRP**			
Mean (SD)	16.3 (12.6)	25.3 (30.9)	19.3 (20.5)
Median [Min, Max]	16.1 [0.0200, 44.9]	13.2 [4.70, 94.0]	14.8 [0.0200, 94.0]
Missing	7 (25.9%)	5 (33.3%)	12 (28.6%)
**IL6**			
Mean (SD)	176 (509)	36.6 (28.6)	116 (387)
Median [Min, Max]	19.0 [3.20, 2030]	27.7 [3.10, 96.3]	22.9 [3.10, 2030]
Missing	11 (40.7%)	3 (20.0%)	14 (33.3%)
**D-Dimer**			
Mean (SD)	1880 (1910)	521 (477)	1400 (1680)
Median [Min, Max]	1030 [220, 6890]	290 [103, 1470]	776 [103, 6890]
Missing	5 (18.5%)	3 (20.0%)	8 (19.0%)
**Fibrinogen**			
Mean (SD)	539 (254)	493 (107)	527 (221)
Median [Min, Max]	554 [179, 1140]	451 [387, 666]	519 [179, 1140]
Missing	14 (51.9%)	10 (66.7%)	24 (57.1%)
**Procalcitonin**			
Mean (SD)	2.57 (5.82)	0.788 (2.54)	1.92 (4.91)
Median [Min, Max]	0.940 [0.0200, 26.6]	0.0500 [0.0200, 8.86]	0.140 [0.0200, 26.6]
Missing	6 (22.2%)	3 (20.0%)	9 (21.4%)
**IgA**			
Mean (SD)	247 (124)	124 (135)	196 (140)
Median [Min, Max]	235 [35.0, 519]	78.5 [11.0, 495]	156 [11.0, 519]
Missing	10 (37.0%)	3 (20.0%)	13 (31.0%)
**IgM**			
Mean (SD)	125 (133)	29.4 (11.5)	95.5 (119)
Median [Min, Max]	73.0 [29.0, 580]	25.5 [21.0, 53.0]	62.5 [21.0, 580]
Missing	9 (33.3%)	7 (46.7%)	16 (38.1%)
**IgG**			
Mean (SD)	953 (413)	631 (315)	822 (404)
Median [Min, Max]	991 [245, 1780]	662 [149, 1290]	771 [149, 1780]
Missing	8 (29.6%)	2 (13.3%)	10 (23.8%)
**Antiviral therapy**			
Remdesivir (10 mg)	4 (14.8%)	9 (60.0%)	13 (31.0%)
Remdesivir (5 mg)	7 (25.9%)	3 (20.0%)	10 (23.8%)
No treatment	11 (40.7%)	1 (6.7%)	12 (28.6%)
Molnupiravir	1 (3.7%)	0 (0%)	1 (2.4%)
Missing	4 (14.8%)	2 (13.3%)	6 (14.3%)
**COVID-19 vaccine**			
Not vaccinated	12 (44.4%)	4 (26.7%)	16 (38.1%)
Two doses	5 (18.5%)	1 (3.7%)	6 (14.3%)
Three doses	8 (29.6%)	10 (66.7%)	18 (42.8%)
Four doses	2 (7.4%)	-	2 (4.8%)

*C-reactive protein (CRP); Interleukin-6 (IL6); Standard deviation (SD).*

**Table 2:** Pathologies of COVID-19 patients treated with tixagevimab–cilgavimab.

Diseases	Group A	Group B
***Cardiovascular disorders (n = 8)***		
Hypertensive cardiopathy	3	-
Atrial fibrillation	2	-
Arterial hypertension	1	-
Ischemic cardiopathy	1	-
Stroke	1	-
***Degenerative diseases (n = 7)***		
Wagner syndrome	1	-
Alzheimer’s disease	4	-
Multiple sclerosis	1	-
Creutzfeldt-Jakob disease	1	-
***Solid tumors (n = 5)***		
Lung carcinoma	4	-
Breast carcinoma	1	-
**Infections (n = 4)**		
Cirrhosis HBV related	1	-
Cryptococcal meningitis	1	-
HIV	2	-
***Autoimmune disorders (n = 3)***		
Autoimmune gastritis	1	-
Rheumatoid arthritis	1	-
Magic Syndrome	1	-
***Other (n = 5)***		
Iatrogenic marrow aplasia	1	-
Chronic kidney disease	4	-
***Oncohematological diseases (n = 15)***		
Chronic lymphocytic leukemia	-	4
Non-Hodgkin lymphoma	-	11

**Table 3:** HR chest CT scan of 42 patients with SARS-CoV-2 infection before treatment with tixagevimab-cilgavimab.

**Group A**
Patient 2: GGO + consolidation
Patient 3: 7/20 + acinetobacter multi-drug-resistant
Patient 4: 15/20
Patient 7: 5/20
Patient 14: GGO
Patient 15: GGO + effusion
Patient 16: 9/20
Patient 18: GGO + thickening
Patient 19: GGO + effusion
Patient 20: Not available
Patient 21: GGO + thickening
Patient 23: no pneumonia
Patient 24: GGO
Patient 27: GGO + thickening
Patient 28: GGO
Patient 29: 13/20
Patient 30: Not available
Patient 31: 7/20
Patient 32: GGO + thickening
Patient 33: GGO
Patient 34: no pneumonia
Patient 35: Not available
Patients 36: Not available
Patient 37: GGO + thickening + effusion
Patient 38: Not available
Patient 39: GGO + thickening + effusion
Patient 40: GGO + thickening
**Group B**
Patient 1: GGO + Legionella infection
Patient 5: 13/20
Patient 6: areoles
Patient 8: Not available
Patient 9: GGO
Patient 10: 18/25
Patient 11: 12/20
Patient 12: GGO
Patient 13: GGO + consolidation + effusion
Patient 17: GGO + thickening
Patient 22: cerebral edema, no pneumonia
Patient 25: GGO
Patient 26: GGO + consolidation
Patient 41: 4/20 + thickening
Patient 42: bilateral GGO

*Ground-glass opacity (GGO).*

## Data Availability

Data is contained within the article.
